# Glycemic Variability in Diabetes Increases the Severity of Influenza

**DOI:** 10.1128/mBio.02841-19

**Published:** 2020-03-24

**Authors:** Rebecca J. Marshall, Pornthida Armart, Katina D. Hulme, Keng Yih Chew, Alexandra C. Brown, Philip M. Hansbro, Conor J. Bloxham, Melanie Flint, Katharina Ronacher, Helle Bielefeldt-Ohmann, Linda A. Gallo, Kirsty R. Short

**Affiliations:** a School of Chemistry and Molecular Biosciences, The University of Queensland, St. Lucia, QLD, Australia; b Priority Research Centre for Healthy Lungs, Hunter Medical Research Institute, New Lambton, NSW, Australia; c The University of Newcastle, Callaghan, NSW, Australia; d Centre for Inflammation, Centenary Institute, Sydney, NSW, Australia; e University of Technology Sydney, Faculty of Science, Ultimo, NSW, Australia; f School of Biomedical Sciences, The University of Queensland, St. Lucia, QLD, Australia; g Mater Research Institute, Translational Research Institute, The University of Queensland, Woolloongabba, QLD, Australia; h Australian Infectious Diseases Research Centre, The University of Queensland, St. Lucia, QLD, Australia; University of Hong Kong

**Keywords:** blood glucose, diabetes, influenza

## Abstract

People with diabetes are two times more likely to die from influenza than people with no underlying medical condition. The mechanisms underlying this susceptibility are poorly understood. In healthy individuals, small and short-lived postprandial peaks in blood glucose levels occur. In diabetes mellitus, these fluctuations become greater and more frequent. This glycemic variability is associated with oxidative stress and hyperinflammation. However, the contribution of glycemic variability to the pathogenesis of influenza A virus (IAV) has not been explored. Here, we used an *in vitro* model of the pulmonary epithelial-endothelial barrier and novel murine models to investigate the role of glycemic variability in influenza severity. *In vitro*, a history of glycemic variability significantly increased influenza-driven cell death and destruction of the epithelial-endothelial barrier. *In vivo*, influenza virus-infected mice with a history of glycemic variability lost significantly more body weight than mice with constant blood glucose levels. This increased disease severity was associated with markers of oxidative stress and hyperinflammation both *in vitro* and *in vivo*. Together, these results provide the first indication that glycemic variability may help drive the increased risk of severe influenza in people with diabetes mellitus.

## Abstract (precis)

Every winter, people with diabetes are at increased risk of severe influenza. At present, the mechanisms that cause this increased susceptibility are unclear. Here, we show that the fluctuations in blood glucose levels common in people with diabetes are associated with severe influenza. These data suggest that glycemic stability could become a greater clinical priority for patients with diabetes during outbreaks of influenza.

## INTRODUCTION

Influenza A virus (IAV) can cause severe disease in people with certain underlying chronic medical conditions. Individuals with diabetes represent one such risk group (1, 2). Specifically, diabetes triples the risk of hospitalization with influenza, quadruples the risk of admission to the intensive care unit, and doubles the risk of a fatal outcome (1–3). Consistent with these clinical observations, murine models demonstrate that following infection with either seasonal or highly pathogenic influenza virus strains, diabetic mice have more severe influenza than healthy mice (4–7).

The increased susceptibility of type 1 (T1) and type 2 (T2) diabetes patients to influenza-associated complications has been associated with the detrimental physiological effects of hyperglycemia (4, 8). However, in the context of the macro- and microvascular complications of diabetes (e.g., heart and kidney disease), there is now a growing body of evidence that glucose variability may have an even greater effect than hyperglycemia *per se* on physiological dysfunction (9, 10).

In healthy individuals, blood glucose levels are maintained within a narrow range (4.4 to 6.7 mmol/liter), including small and short-lived postprandial peaks (9). In people living with diabetes, blood glucose fluctuations are generally greater and more frequent. Relative to the endothelial function seen during steady-state hyperglycemia, endothelial cells exposed to glucose oscillations have an increased propensity for apoptosis and expression of adhesion molecules, high-mobility group box 1 (HMGB1), interleukin-8 (IL-8), nuclear factor κB (NF-κB), and E-selectin (11–14). These oscillation-induced changes in endothelial function are associated with increased oxidative stress (11–14) Specifically, people living with diabetes who have oscillating blood glucose levels and endothelial dysfunction have higher oxidative stress than diabetes patients with steady-state glucose levels (11).

Interestingly, oxidative stress, endothelial cell apoptosis, and endothelial cytokine production are all known to increase the severity of influenza (15–19). During IAV infection, endothelial cells are key drivers of the cytokine storm that underlies much of the observed immunopathology (15, 18, 20). Excessive oxidative stress is also a key driver of lung lesions during severe influenza (21). Oxidative stress can further impair antiviral CD8^+^ T cell responses (22). This effect is most pronounced with memory T cells (22, 23). These data suggest that glycemic oscillations may increase the severity of influenza in persons living with diabetes by altering endothelial cell function and/or affecting antiviral immunity.

At present, research into the effect of glycemic oscillations on influenza severity is limited by the lack of an appropriate animal model of glycemic variability. Glycemic spikes have been studied in mice through repeated administration of a glucose/maltose bolus (24–27). Alternatively, glycemic variability has been modeled using glucose injections in rats fed a high-fat diet (HFD) (28). However, the aforementioned studies failed to control for the confounding effect of increasing the total amount of glucose that an animal is exposed to. Without a direct comparison to mice exposed to the same total glucose levels (with minimal variability), the effect of glycemic oscillations *per se* cannot be confirmed.

Here, we used our previously established *in vitro* model of the pulmonary epithelial-endothelial cell barrier (17, 29) and novel murine models to demonstrate that glycemic variability increases the severity of both a primary infection and a secondary infection with IAV. This increased disease severity was associated with increased pulmonary inflammation and markers of oxidative stress. Given that global rates of diabetes mellitus are increasing, understanding how diabetes contributes to increased influenza severity has an important role in pandemic preparedness.

## RESULTS

### A history of glucose variability increases IAV-induced barrier damage *in vitro*.

**FIG 1:**
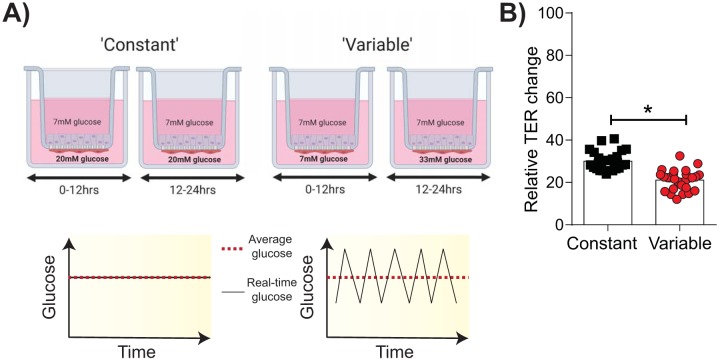
Glycemic variability increases IAV-induced damage of the epithelial-endothelial barrier *in vitro*. (A) Schematic representation of the *in vitro* system used to model constant and variable blood glucose levels. Image created with BioRender. (B) Transepithelial electrical resistance (TER) 24 h after infection with IAV [A/Solomon Islands/03/2006(H1N1)]. Data are expressed relative to those for both the specific well’s baseline TER (i.e., prior to infection) and the mock-inoculated wells for the respective treatment groups. Data were pooled from three independent experiments, and the mean ± SEM is shown. Statistical significance was determined using a Mann-Whitney test. Statistical significance is indicated (*, *P* < 0.05).

To determine whether endothelial exposure to glucose variability could lead to more severe influenza, the effect of glucose variability on an established *in vitro* model of the epithelial-endothelial respiratory barrier was assessed (17, 29). This *in vitro* model reflects the distal lung region where epithelial cells are covered in a fluid layer, called the alveolar lining fluid (30). Accordingly, the cells are cultured in medium instead of at an air-liquid interface, unlike cells in *in vitro* models of the upper respiratory tract (31). Endothelial cells in this model are exposed to either 20 mM glucose (constant conditions) or glucose levels that alternate between 7 mM and 33 mM glucose every 12 h (variable conditions). Importantly, under both constant and variable conditions, endothelial cells are exposed to the same total amount of glucose over the treatment period (Fig. 1A). Following 4 to 5 days of glucose treatment, IAV (H1N1) was added to the upper compartment of the coculture and the transepithelial electrical resistance (TER; a measure of barrier integrity) was assessed over time. At the time of IAV infection, there was no marked difference in the TER of cocultures under variable and constant conditions (see Fig. S1 in the supplemental material). However, at 24 h post-IAV inoculation, the TER under the variable condition was significantly lower than that under the constant condition, indicative of greater barrier damage (Fig. 1B).

10.1128/mBio.02841-19.2 FIG S1 TER, tight junctions, proinflammatory cytokines, and 4HNE adducts of *in vitro* cocultures under variable and constant conditions prior to influenza virus infection. (A) Transepithelial electrical resistance (TER). (B) Staining of *in vitro* cocultures under variable and constant glucose conditions for JAM-1 and Claudin 4 prior to influenza virus infection. Nuclear staining is shown in blue, while junctional staining is shown in green. (C and D) Proinflammatory cytokines (C) and 4HNE adducts (D) in the supernatant of *in vitro* cocultures under variable and constant glucose conditions just prior to influenza virus infection. Data were pooled from three independent experiments, and the mean ± SEM is shown. Statistical significance was determined using a Mann-Whitney test and is indicated (*, *P* < 0.05). Download FIG S1, TIF file, 1.5 MB. Copyright © 2020 Marshall et al. 2020 Marshall et al. This content is distributed under the terms of the Creative Commons Attribution 4.0 International license.

**FIG 2:**
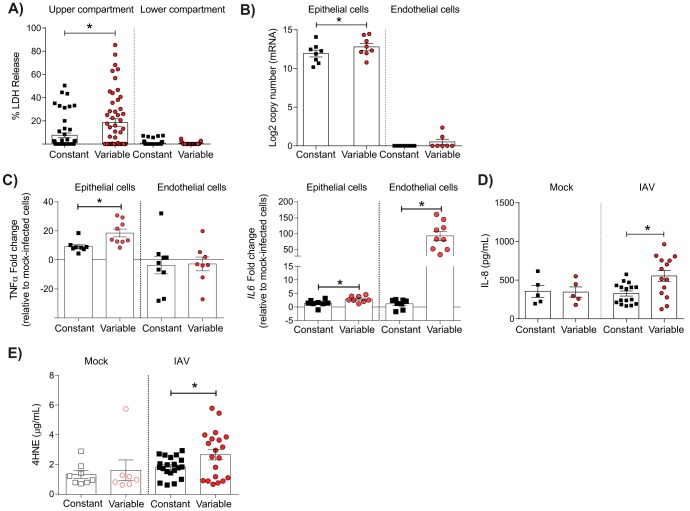
Glycemic variability is associated with IAV-induced cell death, viral replication, inflammation, and oxidative stress *in vitro*. (A) Lactose dehydrogenase levels in the upper and lower compartments of cocultures at 24 h post-IAV infection. Percent LDH release was calculated relative to the level for mock-infected cells in each treatment group (defined as 0%). (B) IAV mRNA copy number in epithelial and endothelial cells at 24 h post-IAV infection. (C) Expression of the genes for TNF-α (left) and IL-8 (right) at 24 h post-IAV infection. Fold change was calculated using the ΔΔ*C_T_* method and is expressed relative to the value for the mock-infected controls. (D) IL-8 concentration in the lower compartment of the coculture at 24 h post-IAV infection. (E) Release of 4-hydroxynonena (4HNE)-protein adducts in the lower compartment of cocultures at 24 h post-mock or IAV infection. Data were pooled from a minimum of three independent experiments, and the mean ± SEM is shown. Statistical significance was determined using a Student’s unpaired *t* test (for data that were normally distributed) or a Mann-Whitney test (for data that were not normally distributed). Statistical significance is indicated (*, *P* < 0.05).

To determine the possible causes of the observed barrier damage, cell death and viral replication were assessed in the coculture system. To assess cell death, the release of lactate dehydrogenase (LDH) in the cell culture supernatant was measured at 24 h post-IAV inoculation. There were significantly higher LDH levels in the upper compartment of infected cocultures with a history of glucose variability than in those with constant, elevated glucose levels (Fig. 2A). In contrast, no difference was observed in the lower compartment of the coculture system (Fig. 2A). Consistent with these data, there was significantly more IAV mRNA in epithelial cells from cocultures that had a history of glucose variability than in those from cocultures with constant, elevated glucose levels (Fig. 2B). Taken together, these data suggest that a history of glucose variability increases IAV replication and that this is associated with increased epithelial cell death.

### A history of glucose variability increases IAV-induced inflammation and oxidative stress *in vitro*.

Glucose variability has previously been associated with increased inflammatory responses (32). To determine if this occurred in the context of IAV infection, expression of the genes for proinflammatory mediators in endothelial and epithelial cells was quantified at 24 h post-IAV inoculation. Prior to IAV infection, there was no significant difference in the expression of genes for a variety of different proinflammatory cytokines between cocultures under constant and variable conditions (Fig. S1). In contrast, at 24 h after IAV infection, the gene for tumor necrosis factor alpha (TNF-α) was significantly upregulated in infected epithelial cells under the variable condition (Fig. 2C). Similarly, there was a significant upregulation of the gene for IL-6 in both epithelial and endothelial cells in cocultures under the variable condition (Fig. 2C), and increased levels of IL-8 were also detected in the supernatant of infected cocultures under the variable condition (Fig. 2D). Increased inflammation in glycemic variability has previously been associated with the induction of oxidative stress (11–14). Consistent with these observations, a history of glycemic variability was associated with the increased production of 4-hydroxynonenal (4HNE) (Fig. 2E), a well-known by-product of lipid peroxidation and a stable marker of oxidative stress. In contrast, prior to IAV infection there were no significant differences in the levels of 4HNE adducts in the supernatants of the cocultures under variable and constant conditions (Fig. S1).

### Establishing a novel murine model of glucose variability.

**FIG 3:**
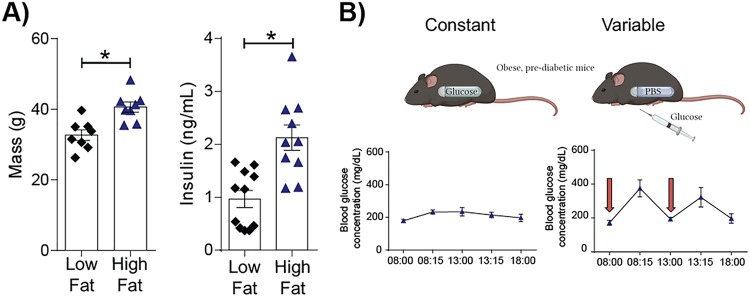
A novel murine model of glycemic variability. (A) Body weight (left) and serum insulin levels (right) of mice on a high-fat or a low-fat diet for 10 weeks. Data were pooled from a minimum of two independent experiments (the mean ± SEM is shown). Statistical significance was determined using a Student’s unpaired *t* test (for data that were normally distributed) or a Mann-Whitney test (for data that were not normally distributed). Statistical significance is indicated (*, *P* < 0.05). (B) The 10-h blood glucose profile in mice implanted with a continuous glucose pump (left) or a pump loaded with PBS (right). Mice with a PBS pump (i.e., mice under the variable glucose condition) were administered twice-daily glucose injections i.p. (the timing is indicated by a red arrow). Data were pooled from two independent experiments, with data points representing mean blood glucose levels ± SEM (3 mice per group).

The results presented above indicate that, *in vitro*, variable glucose levels increase barrier damage, viral titers, oxidative stress, and inflammation. To confirm these data in a more complex *in vivo* system, a novel murine model of glucose variability was established. C57BL/6 mice were fed a high-fat diet (HFD) or a low-fat diet (LFD) for 10 weeks. After 10 weeks on these diets, mice fed the HFD showed key features of prediabetes. These included significantly higher body weights (Fig. 3A) and significantly higher fasting insulin levels (Fig. 3A). Accordingly, mice receiving the HFD were used for all subsequent experiments. To model glucose variability, mice receiving the HFD were intraperitoneally (i.p.) implanted with phosphate-buffered saline (PBS)-loaded Alzet osmotic minipumps and administered glucose i.p. twice daily. This resulted in clear spikes in the intraday blood glucose levels (Fig. 3B). These spikes in blood glucose levels lasted between 2 and 2.5 h (data not shown). In control mice or mice with constant elevated glucose levels receiving the HFD, glucose-loaded Alzet osmotic minipumps were surgically implanted in the peritoneum. This pump releases a constant amount of glucose (0.5 μl/h), such that these mice do not experience marked diurnal or nocturnal intraday glycemic variation (Fig. 3B). Importantly, mice with both constant and variable glucose levels received approximately the same amount of glucose throughout the experimental period. Consistent with this notion, at the end of the experimental period, the average blood glucose concentration (as determined by the glycosylated hemoglobin [HbA1c] concentration) was not significantly different between the two treatment groups (mice with variable glucose levels, 4.66% ± 0.1%; mice with constant glucose levels, 4.57% ± 0.3%).

### Glycemic variability increases the severity of a primary IAV infection.

**FIG 4:**
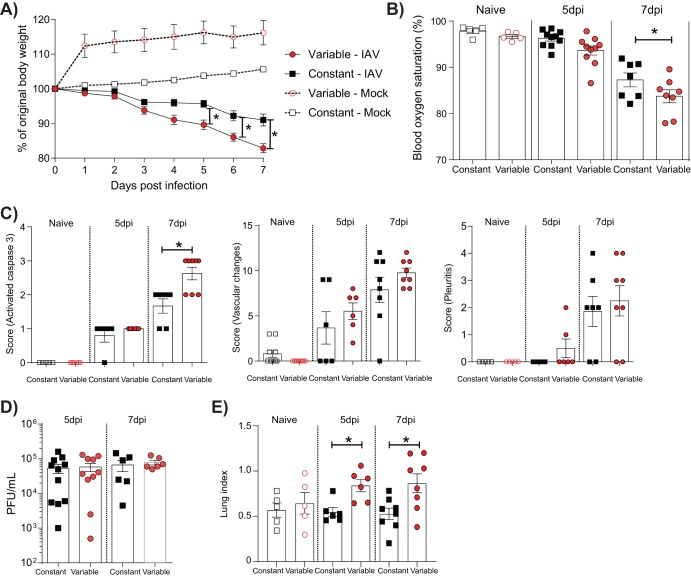
Glycemic variability increases the severity of IAV infection *in vivo*. (A) Percent weight loss of IAV- or mock-inoculated mice with a history of variable or constant blood glucose levels. Weights are displayed as the percentage of the weight at the time of inoculation. (B) Percent blood oxygen saturation of IAV-infected and uninfected mice on various days postinfection (dpi). (C) Histopathology scoring of lung sections for activated caspase 3 (left), vascular changes (middle), and pleuritis (right). (D) Pulmonary viral titers of mice with constant or variable blood glucose levels. (E) Lung index in mice with constant or variable blood glucose levels. The lung index was calculated as [lung weight (in grams)/body weight (in grams)] × 100. Data were pooled from a minimum of two independent experiments, and the mean ± SEM is shown. Statistical significance was determined using a 2-way analysis of variance with Tukey’s *post hoc* test or a Student’s unpaired *t* test (for data that were normally distributed), or a Mann-Whitney test (for data that were not normally distributed). Statistical significance is indicated (*, *P* < 0.05).

After 15 days of glucose treatment, mice were inoculated with IAV or mock inoculated and disease severity was assessed. IAV-infected mice under the variable condition lost significantly more body weight over the course of the infection (Fig. 4A) and had lower blood oxygen saturation at 7 days postinoculation than infected mice under the constant condition (Fig. 4B). In contrast, mock-infected mice under the constant and variable conditions did not lose any weight over the time course and showed no difference in the levels of blood oxygen saturation. IAV-infected mice in the variable glucose condition group also had significantly higher pulmonary levels of activated caspase 3 expression at 7 days postinfection (dpi) than infected mice in the constant glucose condition group (Fig. 4C). In contrast, no difference in the severity of vascular changes, the rate of pleuritis, or pulmonary viral titers was observed between the two treatment groups. Consistent with the increased inflammation in the variable glucose condition group, infected mice in the variable glucose condition group had a significantly higher lung index than mice in the constant glucose condition group at both 5 and 7 dpi.

**FIG 5:**
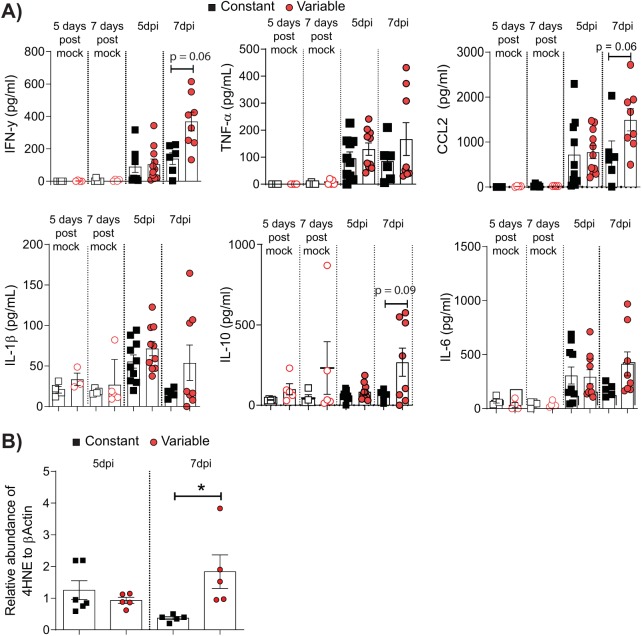
Glycemic variability increases IAV-induced pulmonary inflammation and oxidative stress *in vivo*. (A) Cytokines in lung homogenates at various days postinfection (dpi). (B) The presence of 4-hydroxynonenal (4HNE)-protein adducts in the lung homogenates of IAV-inoculated mice detected by immunoblotting. Data are expressed relative to the β-actin levels detected in the same sample. Data were pooled from a minimum of two independent experiments, and the mean ± SEM is shown. Statistical significance was determined using a Student’s unpaired *t* test (for data that were normally distributed) or a Mann-Whitney test (for data that were not normally distributed). Statistical significance is indicated (*, *P* < 0.05).

*In vitro*, glucose variability was associated with an increased proinflammatory and oxidative stress response to IAV (Fig. 2). To determine if a similar pattern was observed *in vivo*, proinflammatory cytokines in the lung were assessed in all mice after IAV or mock inoculation. IAV-infected mice in the variable glucose condition group had significantly higher levels of gamma interferon (IFN-γ) in the lung at 7 dpi than IAV-infected mice in the constant glucose condition group (Fig. 5A). This was accompanied by a trend toward increased TNF-α, CCL2, IL-1β, IL-10, and IL-6 levels in the lungs of infected mice in the variable glucose condition group at 7 dpi, although this difference did not reach statistical significance. The increased proinflammatory responses in mice in the variable glucose condition group were associated with a significant increase in pulmonary levels of 4HNE, suggestive of increased oxidative stress (Fig. 5B).

### Glycemic variability increases the severity of a secondary IAV infection.

**FIG 6:**
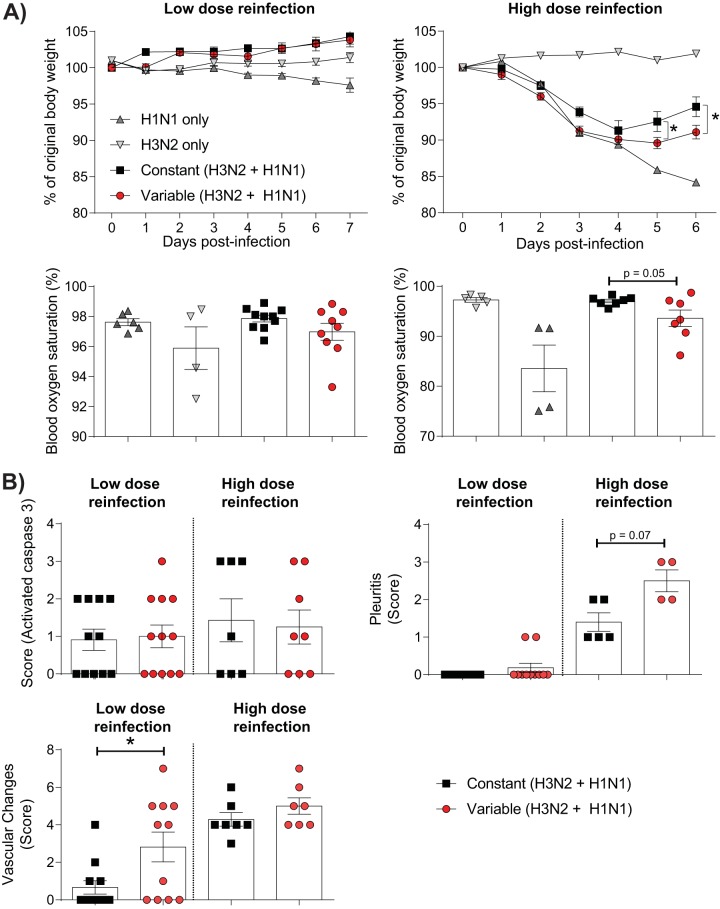
Glycemic variability increases the severity of IAV infection *in vivo* after reinfection. (A) (Top) Percent weight loss of IAV- or mock-inoculated mice with a history of variable or constant blood glucose levels. Weights are displayed as a percentage of the weight at the time of inoculation. Mice were reinfected with either a low (1,000-PFU) or a high (100,000-PFU) dose of A/PR/8(H1N1). (Bottom) Percent blood oxygen saturation of IAV-infected mice at 7 (low dose) or 6 (high dose) days after reinfection. (B) Histopathology scoring of lung sections for activated caspase 3, vascular changes, and pleuritis at 7 (low dose) or 6 (high dose) days after reinfection. Statistical significance was determined using a 2-way analysis of variance with Tukey’s *post hoc* test or a Student’s unpaired *t* test (for data that were normally distributed) or a Mann-Whitney test (for data that were not normally distributed).

The murine model described above best reflects individuals in whom there has been no previous exposure to IAV. However, given that the vast majority of individuals have antibodies to influenza virus by the age of 6 years (33), this may not represent the majority of the human population. We therefore sought to assess whether glycemic variability increased the severity of IAV in mice with a history of exposure to influenza virus (Fig. S2). Accordingly, obese, prediabetic mice were infected with A/Aichi/68 X-31(H3N2) [HKx31(H3N2)] and allowed to recover from the infection for 2 weeks. The mice were then subjected to variable or constant glucose conditions as described above. After 2 weeks of glucose treatment, mice were infected with A/Puerto Rico/8/34(H1N1) [A/PR/8(H1N1)]. Importantly, while HKx31(H3N2) has all the same internal genes as A/PR/8(H1N1), HKx31(H3N2) has antigenically distinct surface proteins. Accordingly, antibodies developed to HKx31(H3N2) do not protect against reinfection with A/PR/8(H1N1) (34). Following reinfection with 10^5^ PFU of A/PR/8(H1N1), all mice lost body weight relative to mice infected with HKx31(H3N2) alone (Fig. 6A). While nonimmune mice [i.e., those not previously infected with HKx31(H3N2)] continued to lose weight throughout the infection period, reinfected mice began to regain body weight at 5 dpi. This finding is consistent with the presence of cross-protective CD8^+^ T cells in these mice (34). Strikingly, mice in the constant glucose condition group regained significantly more body weight after A/PR/8(H1N1) reinfection than reinfected mice in the variable glucose condition group (Fig. 6A). This increased weight gain was associated with a trend toward increased blood oxygen saturation in reinfected mice in the constant glucose condition group, although this difference fell just short of statistical significance (*P* = 0.05). In contrast, reinfection with a lower dose of A/PR/8(H1N1) (10^3^ PFU) resulted in limited clinical signs (Fig. 6A).

10.1128/mBio.02841-19.3 FIG S2 Schematic representation of the murine model of glycemic variability with preexisting immunity. Created with BioRender. Download FIG S2, TIF file, 0.1 MB. Copyright © 2020 Marshall et al. 2020 Marshall et al. This content is distributed under the terms of the Creative Commons Attribution 4.0 International license.

**FIG 7:**
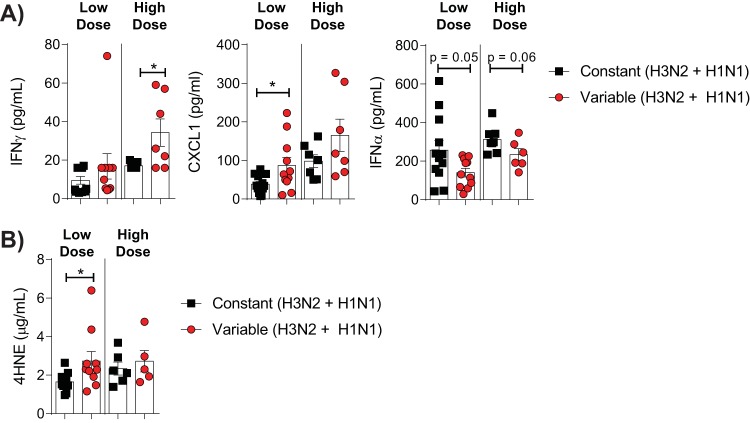
Glycemic variability increases IAV-induced pulmonary inflammation and oxidative stress after reinfection. (A) Cytokines in lung homogenates of mice reinfected with either a low (1,000-PFU) or a high (100,000-PFU) dose of A/PR/8(H1N1). (B) Concentration of 4-hydroxynonenal (4HNE)-protein adducts in the lung homogenates of IAV-infected mice. All data were obtained at either 7 (low dose) or 6 (high dose) days of infection. Data were pooled from a minimum of two independent experiments, and the mean ± SEM is shown. Statistical significance was determined using a Student’s unpaired *t* test (for data that were normally distributed) or a Mann-Whitney test (for data that were not normally distributed). Statistical significance is indicated (*, *P* < 0.05).

In contrast to our observations in the model of primary IAV infection, reinfected mice in the variable glucose condition group had no significant increase in pulmonary caspase 3 levels relative to mice with constant blood glucose levels (Fig. 6B). However, mice in the variable glucose condition group reinfected with a low dose of A/PR/8(H1N1) had a significant increase in pulmonary vascular changes relative to mice in the constant glucose condition group, while mice in the variable glucose condition group reinfected with a high dose of A/PR/8(H1N1) had a trend toward increased pleuritis relative to mice in the constant glucose condition group (Fig. 6B). These changes were associated with increased levels of IFN-γ (high-dose reinfection), CXCL1 (low-dose reinfection), and reduced levels of IFN-α in both the low-dose (*P* = 0.05) and the high-dose (*P* = 0.06) reinfection groups (Fig. 7A). In the low-dose reinfection group, these changes were associated with an increase in the level of 4HNE and oxidative stress in the lungs of infected mice with glycemic variability (Fig. 7B). Together, these data suggest that even in the presence of preexisting immunity, glycemic variability is associated with increased IAV severity.

## DISCUSSION

It is well documented that diabetes confers an increased risk for severe influenza, yet the mechanisms that underlie this susceptibility are largely unclear (7). Here, a previously described *in vitro* model of the epithelial-endothelial respiratory barrier was used along with novel murine models of glycemic variability to provide the first evidence that glycemic variability can increase influenza severity.

In the present study, glycemic variability drove numerous changes *in vitro* and *in vivo* during IAV infection. These changes provide some insight into the possible mechanisms of increased IAV severity in the context of glycemic variability. Glycemic variability and increased influenza severity were associated, both *in vitro* and *in vivo*, with increased levels of 4HNE adducts, a known marker of oxidative stress. While this remains a purely correlative association and requires more in-depth investigation (including measuring other markers of oxidative stress), these findings are consistent with previous observations that a key outcome of glycemic variability is the overproduction of reactive oxygen species, which, in turn, drives increased endothelial production of inflammatory mediators (35, 36). Indeed, in the present study, the increased production of proinflammatory mediators under the variable condition was observed both *in vivo* and *in vitro*, and this was associated with more severe influenza. Should these data be extrapolated to the human population, it raises the intriguing possibility that the long-term administration of oxidative stress inhibitors (i.e., prior to influenza virus infection) could be a viable therapeutic approach to reduce the burden of influenza in persons living with diabetes with glycemic variability. This is consistent with the findings of previous studies showing that long-term treatment with *N*-acetylcysteine (an antioxidant) reduced the severity of influenza-like symptoms in people with nonrespiratory chronic degenerative diseases (including metabolic conditions) (37).

Importantly, in the experimental models employed in the present study, glucose treatments were stopped before IAV infection, such that cells and mice were not exposed to experimentally induced glucose fluctuations during infection. This experimental design was chosen in light of previous studies showing that the exogenous administration of glucose during IAV infection alters viral pathogenesis (38). However, the observation of increased influenza severity in cells and mice with a history of glycemic variability suggests that the detrimental effects of glycemic variability extend beyond the period of variability itself. This observation is similar to that made in previous studies, where the vascular benefits of intensive glucose therapy long outlasted the experimental period. This phenomenon is termed the “legacy effect” of intensive glycemic control (39–41). The mechanisms explaining the legacy effect are unclear, but it has been proposed that epigenetic changes influencing the regulation of NF-κB transcription occur and persist even after transient hyperglycemia (42–44). The production and accumulation of advanced glycation end products during periods of poor glycemic control may also persist beyond periods of hyperglycemia and induce sustained cellular changes (45–47). In clinical studies, the legacy effect has been observed to endure for more than 10 years after the experimental period has finished (39). It is therefore plausible that a legacy effect of glycemic variability would persist after a longer delay between glucose treatment and IAV infection in the *in vivo* models presented herein. It is crucial for future studies to investigate whether any putative legacy effect could be reversed with subsequent and sustained periods of minimal glycemic variability. The existence of a legacy effect, if confirmed *in vivo*, gives gravity to the importance of early and sustained glycemic control to mitigate lasting consequences for an individual’s susceptibility to influenza.

This study developed two novel murine models of glycemic variability, the first one for a primary IAV infection and the second one for reinfection with a heterologous IAV strain. To the best of our knowledge, these are the first *in vivo* models of glycemic variability that controlled the total glucose exposure between treatment groups. Thus, it is possible to conclude that the effects of glycemic variability on IAV pathogenesis were due to variations in blood glucose levels rather than an overall increase in glucose exposure. Nevertheless, there were still several limitations inherent in the murine models developed. First, the periods of glycemic variability in the present study were limited to 14 days, as this aligned with the maximum *in vivo* functionality of the glucose pump used in the control group. In people living with diabetes, periods of glycemic variability are likely to extend over a much longer period of time. This model therefore does not reflect the complex physiological changes that occur in an individual who has been subject to years of glycemic variability (e.g., due to undiagnosed T2 diabetes). Second, it is important to acknowledge that mice in the constant glucose condition group were not injected with PBS twice daily (which would be the standard control for the glucose injections that were performed in the variable glucose condition group). This decision was made because physiological stressors (such as twice-daily i.p. injections) can cause transient spikes in blood glucose levels, and this side effect was considered undesirable in the constant glucose condition group. However, any confounding influence that this experimental design may have had on the data would still not be sufficient to explain our *in vitro* observations that glucose variability increased the severity of influenza. Finally, it must be acknowledged that murine models of disease can never truly depict the complexity of disease that occurs in humans. Thus, while the findings of this study have been confirmed both *in vitro* and *in vivo*, it is necessary to validate these findings in people living with diabetes with poor glycemic control.

Importantly, the global incidence of both T1 and T2 diabetes is on the rise (48). This is coupled with a growing recognition that diabetes increases the severity of a wide variety of different infectious diseases, including influenza (49). These global trends should emphasize the need for further research in this area, while also providing the impetus to improve public awareness that influenza virus vaccination is of the upmost importance for people living with diabetes (3).

## MATERIALS AND METHODS

### Cells.

NCI-H441 cells were obtained from the American Type Culture Collection (ATCC; Manassas, VA) and cultured in RPMI medium (Gibco, Grand Island, NE) with 10% fetal bovine serum (FBS; Gibco) and 1% penicillin-streptomycin (Gibco). NCI-H441 cells were used between passages 2 and 14. Primary human pulmonary microvascular endothelial cells (HPMECs) were obtained from Sciencell (Carlsbad, CA) and cultured in endothelial cell growth medium (Sciencell). HPMECs were used at passages 2 to 7. Madin-Darby canine kidney (MDCK) cells were obtained from ATCC and kept in Dulbecco modified Eagle medium (DMEM; Gibco) with 10% FBS and 1% penicillin-streptomycin. MDCK cells were used between passages 20 and 50. All cell lines were cultured using a humidified 37°C incubator with 95% O_2_ and 5% CO_2_.

### Glucose variability *in vitro*.

A coculture system of NCI-H441 cells and HPMECs was developed essentially as described previously (16). Briefly, Costar HTS Transwell 24-well plates (0.4-μm pore size; Sigma, Kawasaki, Japan) were coated with 0.1 mg/ml of isolated rat tail collagen I (Sigma) in 0.1 M acetic acid (Sigma) overnight at room temperature. Both sides of the membrane were coated. HPMECs were seeded in RPMI (Gibco) with 10% FBS and 1% penicillin-streptomycin (Gibco) at a density of 10 × 10^5^ cells/ml and incubated for 2 h to allow adherence to the membrane. Then, NCI-H441 cells were seeded at a density of 2.5 × 10^5^ cells/ml on the upper side of the membrane in RPMI (Gibco) with 5% FBS and 1% penicillin-streptomycin. Once the cells reached confluence (∼3 to 4 days), the medium was refreshed every 12 h. The upper compartment was refreshed with RPMI containing 5% FBS, 1% penicillin-streptomycin, 1 μM dexamethasone (Sigma), and 7 mM glucose (Gibco), while the lower compartment was refreshed with RPMI (5% FBS, 1% penicillin-streptomycin) and different concentrations of glucose (either 20 mM glucose every 12 h or alternating between 7 mM and 33 mM glucose every 12 h). Importantly, primary endothelial cells still retained their endothelial phenotype after culture in RPMI (data not shown). Once the TER across the membrane reached >1,000 Ω, the cells were infected with IAV.

### Virus strains.

Virus stocks of A/Solomon Islands/03/2006(H1N1) [Solomon Islands/06(H1N1)], A/H1N1/Auckland/09(H1N1), A/Puerto Rico/8/34(H1N1), and A/Aichi/68 X-31(H3N2) were prepared in embryonated chicken eggs, and the titers of infectious virus were determined using three independent plaque assays on MDCK cells as described previously (50).

### *In vitro* virus inoculation.

At 2 h prior to infection, the medium in the upper and lower compartments of the Transwell was refreshed with RPMI (Gibco) with 2% FBS (Gibco) and 1% penicillin-streptomycin (Gibco). A/Solomon Islands/03/2006(H1N1) was then added to the upper compartment at a multiplicity of infection of 5. Alternatively, mock-infected wells received an equivalent volume of naive allantoic fluid (NAF). Virus was not removed after infection, and cells were monitored over time. Infections of all treatment groups were performed in the presence of 12 mM glucose (in both the upper and lower compartments) to mimic the fact that infection is associated with elevated blood glucose levels (51, 52).

### TER.

Transepithelial electrical resistance (TER) was monitored using an EVOM2 voltohmmeter (World Precision Instruments, Sarasota, FL) with an STX2 chopstick electrode.

### LDH assay.

LDH release was measured using a CytoTox 96 nonradioactive cytotoxicity assay (Promega, Mannheim, Germany) according to the manufacturer’s instructions. Data are expressed relative to the amount of LDH released from lysed NCI-H441 cells and HPMECs.

### Mice.

C57BL/6 male mice were obtained from the Animal Resources Centre (ARC; Australia). Mice were fed a high-fat diet *ad libitum* consisting of 40% calories from fat (Specialty Feeds, Glen Forrest, Australia) or a low-fat diet consisting of 12% calories from fat (Specialty Feeds, Glen Forrest, Australia) for 10 weeks and supplied with water *ad libitum*.

### Primary infection with IAV.

After 10 weeks on a high-fat diet, an Alzet osmotic minipump (number model 2002) was implanted in the peritoneum of the mice (Alzet, Cupertino, CA). In the variable glucose treatment group, the pump was loaded with PBS (released at a rate of 0.5 μl/h). These mice then received twice-daily i.p. injections of glucose (10 mg; Phebra, Lane Cove, Australia) starting at 1 day after surgery and continuing for 14 days. In the constant glucose treatment group, the pump was loaded with 1.55-mg/ml glucose (released at a rate of 0.5 μl/h), such that after 15 days, mice in the constant and variable glucose condition groups received approximately the same amount of total glucose (∼300 mg). The mice were then anesthetized with isoflurane using a Stinger Research anesthetic gas machine (Darvall, Payson, AZ) and inoculated intranasally with 10^2^ PFU of influenza A/H1N1/Auckland/09(H1N1) virus. Importantly, glucose was not administered during the infection period, as it has previously been shown that this can alter IAV pathogenesis (38).

### Reinfection model of IAV.

After 10 weeks on the high-fat diet, mice were anesthetized with isoflurane as described above and inoculated intranasally with 1,000 PFU of influenza HKx31(H3N2) virus or PBS. The mice were monitored daily for weight loss and allowed to recover for 14 days. An Alzet osmotic minipump (model number 2002) was then implanted in the peritoneum of select treatment groups as described above. After 15 days of either constant or variable glucose treatment, the mice were then anesthetized and inoculated intranasally with either (i) 10^3^ PFU of PR8(H1N1), (ii) 10^5^ PFU of PR8(H1N1), or (iii) PBS. The mice were monitored daily for weight loss and clinical signs of disease.

### Glucose test strip measurements.

A blood sample was obtained from mice using a tail prick, and blood glucose levels were measured using a SensoCard Plus blood glucose monitor with SensoCard test strips (Point of Care Diagnostics, North Rocks, Australia).

### Blood oxygen saturation.

Blood oxygen saturation was measured using a Mouseox Plus collar oximeter (Starr, Oakmont, PA).

### Collection of blood.

After euthanasia, blood was collected from all mice by a cardiac bleed using a 1-ml insulin syringe (Terumo Corporation, Tokyo, Japan). Whole blood was collected and stored in heparin-coated tubes (Greiner Bio-One, Kremsmünster, Austria) at 4°C. Serum was isolated by centrifugation of whole-blood samples and subsequent collection of the supernatant.

### HbA1c.

HbA1c levels in whole blood were measured using a point-of-care (POC) HbA1c analyzer, consisting of a DCA Vantage analyzer (Siemens, Munich, Germany) with DCA Vantage HbA1c reagent cartridges (Siemens). The absorbance was read using a SpectraMax 250 plate reader (Molecular Devices).

### Enumeration of viral load.

The right superior lobe, middle lobe, inferior lobe, and post-caval lobe were collected from each mouse. Lungs were homogenized in DMEM (Gibco) using a Qiagen TissueLyser II apparatus (Qiagen, Hilden, Germany). The homogenate was then centrifuged, and the supernatant was collected and stored at −80°C. Alternatively, cell culture supernatants were collected at the specified time points and stored at −80°C. The viral load was determined by a plaque assay on MDCK cells as described previously (50).

### Histology.

The left lobe of each mouse was inflated by intratracheal administration of PBS (600 μl; Gibco). The left lobe was then fixed in 10% neutral buffered formalin and then transferred to 70% ethanol for processing by the Core Histology Facility, Translational Research Institute, Brisbane, Australia. Samples were embedded in paraffin wax, and sections with a thickness of 5 μm were stained with hematoxylin and eosin. Lungs were assessed for vascular changes, bronchitis, interstitial inflammation, alveolar inflammation, pneumocyte hypertrophy, and pleuritis by a veterinary pathologist who was blind to the study design.

### Lung index.

The lung index was calculated as described by Luo and colleagues (53). Briefly, lungs were weighed postmortem, and the lung index was calculated as [lung weight (in grams)/body weight (in grams)] × 100.

### Immunoblot assays.

The total protein concentration was determined by a bicinchoninic acid (BCA) assay (Thermo Scientific, IL, USA). Samples were boiled for 10 min to induce the denaturation of protein to facilitate the separation of proteins based on size. Protein samples (30 mg) and Precision Plus Protein WesternC standards (Bio-Rad, CA, USA) were resolved on 4 to 15% gradient Mini-Protean TGX stain-free polyacrylamide gels (Bio-Rad) and then transferred onto polyvinylidene difluoride membranes (Merck Millipore, Australia). The blots were blocked with a solution of 5% bovine serum albumin (BSA) in Tris-buffered saline and Tween 20 (T-BST) for 1 h. The blots were then incubated with primary antibody targeting 4HNE (catalog number ab46545; Abcam, Cambridge, MA, USA) in 1% BSA and T-BST overnight, washed, and then incubated with horseradish peroxidase-conjugated anti-rabbit IgG secondary antibody (R&D Systems, Minneapolis, MN, USA). The blots were developed using SuperSignal West Femto maximum-sensitivity substrate (Thermo Scientific) and visualized by chemiluminescence (ChemiDoc imaging system; Bio-Rad) (54).

### Quantification of 4HNE-protein adducts.

The levels of 4HNE in cell culture supernatant and lung homogenate were measured using a lipid peroxidation (4HNE) assay kit (Abcam, Cambridge, MA).

### Serum insulin levels.

Serum insulin levels were determined using an ultrasensitive mouse insulin enzyme-linked immunosorbent assay kit (Crystal Chem, Elk Grove Village, IL) per the manufacturer’s instructions.

### Cytokine levels.

Cytokine concentrations were determined using a mouse or human antiviral response LegendPlex immunoassay according to the manufacturer’s instructions (BioLegend, San Diego, CA).

### RNA extraction and cDNA acid synthesis.

RNA was extracted from cells using an RNeasy plus kit (Qiagen). RNA was isolated from clarified lung homogenates using a Roche High Pure RNA isolation kit (Roche, Basel, Switzerland) according to the manufacturer’s instructions. cDNA was synthesized using a high-capacity cDNA reverse transcription kit (Applied Biosystems) on a Mastercycler thermocycler (Eppendorf, Hamburg, Germany) according to the manufacturer’s instructions using either random primers or oligo(dT) oligonucleotides (Roche).

### qPCR of host genes.

Quantitative PCR (qPCR) was performed using the SYBR green reagent (Applied Biosystems). All primer and probe sequences are listed in Table 1. Reactions with primer efficiencies outside the range of 95 to 105% and/or with multiple peaks detected by melt curve analysis were excluded from subsequent analyses. qPCR conditions were applied according to the manufacturer’s instructions using a 96-well reaction plate (Applied Biosystems) and a QuantStudio (version 6) Flex real-time PCR system (Thermo Fisher Scientific, Waltham, MA). The glyceraldehyde-3-phosphate dehydrogenase (GAPDH) gene was used as a housekeeping gene, and relative gene expression was determined using the comparative cycle threshold (ΔΔ*C_T_*) method.

**TABLE 1:** Primers used in this study

Gene	Orientation^*a*^	Sequence
GAPDH	F	5′-CGAGATCCCTCCAAAATCAA-3′
	R	5′-TTCACACCCATGACGAACAT-3′
IL-6	F	5′-TTCACACCCATGACGAACAT-3′
	R	5′-TTTTCTGCCAGTGCCTCTTT-3′
TNF-α	F	5′-CTCTGCACCCAGTTTTCCTT-3′
	R	5′-TGAGGTACAGGCCCTCTGAT-3′

a F, forward; R, reverse.

### Viral copy number.

Viral copy number was determined as described previously (50).

### Statistical analysis.

GraphPad Prism software (version 6.00) was used for all statistical calculations. Outliers within data sets were excluded using Grubb’s outlier test (55). The normal distribution of the data was assessed using the Shapiro-Wilk test. Data which were not normally distributed were analyzed using the appropriate nonparametric test. A one-sample *t* test was used to determine whether the sample mean was statistically significantly different from the relevant hypothesized population (0 or 100).

### Study approval.

All animal experiments were conducted in accordance with the *Australian Code for the Care and Use of Animals for Scientific Purposes* (56) and approved by the University of Queensland Animal Ethics Committee (permit no. 071/17).

10.1128/mBio.02841-19.1 TEXT S1 Supplemental material and methods. Download Text S1, DOCX file, 0.02 MB. Copyright © 2020 Marshall et al. 2020 Marshall et al. This content is distributed under the terms of the Creative Commons Attribution 4.0 International license.
