# Supplementary figures and images for: Glycemic Variability in Diabetes Increases the Severity of Influenza
Source: mBio. 2020 Mar 24;11(2):e02841-19. doi: 10.1128/mBio.02841-19 (PMC7157527; doi:10.1128/mBio.02841-19)

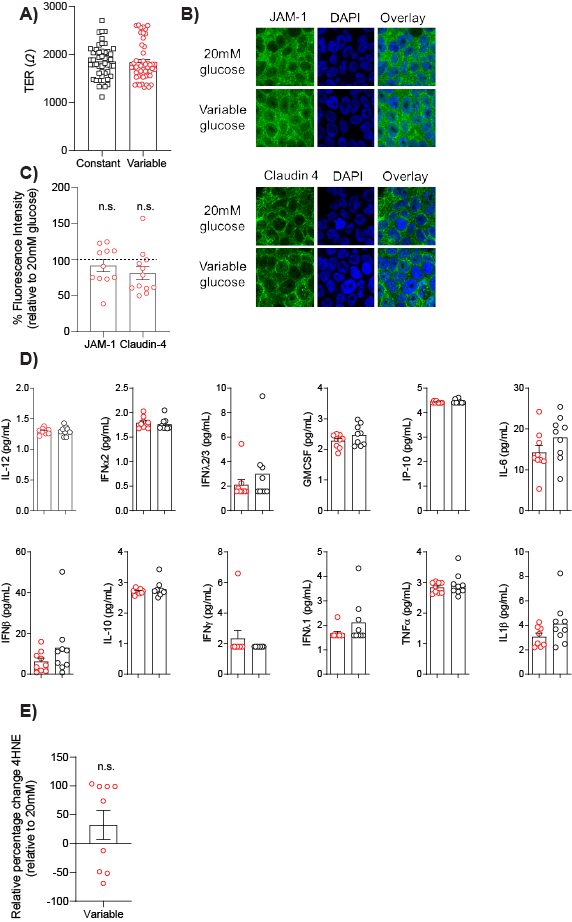

Supplement: FIG S1 [file mBio.02841-19-sf001.tif]

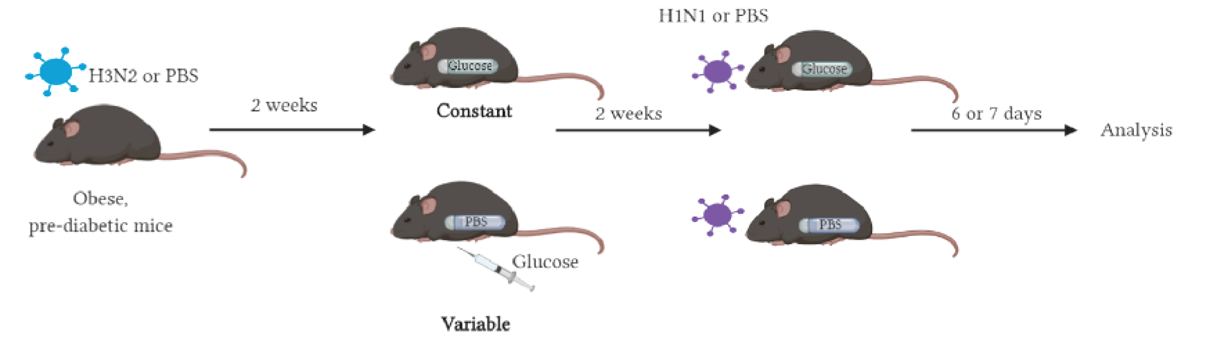

Supplement: FIG S2 [file mBio.02841-19-sf002.tif]
