# Supplementary material for: Glycemic Variability in Diabetes Increases the Severity of Influenza
Source: mBio. 2020 Mar 24;11(2):e02841-19. doi: 10.1128/mBio.02841-19 (PMC7157527; doi:10.1128/mBio.02841-19)
Supplement: TEXT S1 [file mBio.02841-19-s0001.docx]

**SUPPLEMENTAL MATERIAL AND METHODS:**

**Immunofluorescence:** Cells grown on a transwell membrane were fixed, stained and mounted on a glass slide with mounting medium containing 4’,6-diamidino-2-phenylindole (DAPI) (Vector Laboratories, Burlingame, CA, USA) as previously described [1]. Primary antibodies used were α-junctional adhesion molecule 1 (Santa Cruz Biotechnology, Santa Cruz, CA, USA) and α-claudin-4 (Life Technologies, Gaithersburg, MD, USA). Secondary antibodies were Alexa 488 α-mouse IgG2a and Alexa 488 α-rabbit IgG (Life Technologies).

Immunolabeling of transwell membranes were visualised using a Laser Scanning Microscope 710 (LSM 710) (Zeiss, Jena, Germany). Staining intensity of tight junction proteins was quantified using ImageJ software (National Institutes of Health, Bethesda, MD, USA). Specifically, an ImageJ macro was created to measure the mean grey value within a 6cm^2^ area. Each measurement was taken of an image section with 8-12 nuclei sitting within this 6cm^2^ area of transwell confocal images. Three measurements were taken per image, and 3 images were quantified per transwell membrane.

**SUPPLEMENTAL FIGURE LEGEND:**

**Supplemental Figure 1: Barrier integrity of variable and constant co-cultures prior to infection. A)** Trans-epithelial electrical resistance (TER) of variable (7mM glucose/33mM glucose) and constant (20mM glucose) co-cultures prior to infection with IAV. **B)** Representative immunofluorescence images (63x magnification) of apical junction complex proteins of uninfected epithelial cells. Epithelial cells were grown on a transwell membrane in co-culture with endothelial cells exposed to either constant or variable glucose levels. Prior to IAV infection, cells were fixed and the nucleus and the relevant tight junction proteins were stained (blue and green, respectively). **C**): The percentage of fluorescence intensity in epithelial cells from uninfected variable co-cultures relative to uninfected epithelial cells from uninfected constant co-cultures (defined as 100%) prior to IAV infection. **D)** Cytokines in the basolateral supernatant of variable and constant co-cultures prior to IAV infection. **E)** Relative percentage change in the release of 4-hydroxynonena (4HNE)-protein adducts in the lower compartment of variable co-cultures prior to IAV infection. Data is expressed relative to 4HNE-protein adducts in the lower compartment of constant co-cultures prior to IAV infection. All data is pooled from three independent experiments and the mean ± SEM is shown. JAM: junctional adhesion molecule. n.s: not-significant as determined by a one-sample student’s t-test where the hypothetical value is defined as 100 (**C)** or 0 (**E**).

**Supplementary Figure 2: A schematic representation of the murine model of glycaemic variability in the context of pre-existing immunity.**

**REFERENCES:**

1. Short, K.R., et al., *Influenza virus damages the alveolar barrier by disrupting epithelial cell tight junctions.* European Respiratory Journal, 2016: p. ERJ-01282-2015.
